# Cohabiting Plant‐Wearable Sensor In Situ Monitors Water Transport in Plant

**DOI:** 10.1002/advs.202003642

**Published:** 2021-03-09

**Authors:** Yangfan Chai, Chuyi Chen, Xuan Luo, Shijie Zhan, Jongmin Kim, Jikui Luo, Xiaozhi Wang, Zhongyuan Hu, Yibin Ying, Xiangjiang Liu

**Affiliations:** ^1^ College of Biosystems Engineering and Food Science Zhejiang University Hangzhou 310058 China; ^2^ Department of Engineering University of Cambridge Cambridge CB3 0FF UK; ^3^ College of Information Science and Electronic Engineering Zhejiang University Hangzhou 310058 China; ^4^ College of Agriculture and Biotechnology Zhejiang University Hangzhou 310058 China

**Keywords:** electronic tattoos, flexible electronics, phenotyping, sap flow, water allocation

## Abstract

The boom of plant phenotype highlights the need to measure the physiological characteristics of an individual plant. However, continuous real‐time monitoring of a plant's internal physiological status remains challenging using traditional silicon‐based sensor technology, due to the fundamental mismatch between rigid sensors and soft and curved plant surfaces. Here, the first flexible electronic sensing device is reported that can harmlessly cohabitate with the plant and continuously monitor its stem sap flow, a critical plant physiological characteristic for analyzing plant health, water consumption, and nutrient distribution. Due to a special design and the materials chosen, the realized plant‐wearable sensor is thin, soft, lightweight, air/water/light‐permeable, and shows excellent biocompatibility, therefore enabling the sap flow detection in a continuous and non‐destructive manner. The sensor can serve as a noninvasive, high‐throughput, low‐cost toolbox, and holds excellent potentials in phenotyping. Furthermore, the real‐time investigation on stem flow insides watermelon reveals a previously unknown day/night shift pattern of water allocation between fruit and its adjacent branch, which has not been reported before.

## Graphical abstract

A plant‐wearable sensor in situ reveals water transport inside a plant. Due to a special design and the materials chosen, the realized plant‐wearable sensor is thin, soft, lightweight, air/water/light‐permeable, and shows excellent biocompatibility, therefore enabling detection in a continuous and non‐destructive manner.

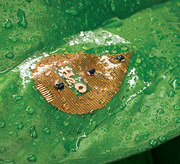

## 1 Introduction

Plant phenotyping, large‐scale approaches to interpret the underlying molecular genetics in conjunction with the biological causes of phenotypic traits of a plant, has revolutionized our understanding of biology for decades.^[^ ^1^, ^2^ ^]^ Plant phenotyping has board importance in both basic and translational research, which is equally relevant to goals as disparate as yield improvement in food and energy crops, global food security, environmental remediation using plants, as well as understanding complex networks that control fundamental life processes that how a single gene or the whole genome translate into the full set of plant phenotypic traits.^[^ ^2^, ^3^, ^4^, ^5^ ^]^ The boom of plant phenotyping highlights the needs for measuring phenotypic traits of an individual plant with sufficient resolutions (in both space and time) and accuracy, such as plant morphologies (e.g., leaf morphology, branching angles, internode lengths, root architectures) and internal physiological traits. Although the physiological traits are more reliable indicators for genetic variance, most phenotyping approaches were based on the morphologies because of their relatively easy assessment by conventional imaging technologies. Rapid and continuous tracking plant's internal physiological traits remain challenges for the current silicon‐based sensing technologies, due to the mechanical mismatch between the traditional rigid sensors and the soft bio‐organism can either cause damage to the plant or intervene its normal physiological process. Therefore, so far, the physiological characteristics were only assessed using destructive or indirect approaches that are often complicated and time/labor‐consuming, far below the requirements for high‐throughput plant phenotyping.^[^ ^6^, ^7^, ^8^ ^]^

New opportunities to address these problems emerge recently owing to the tremendous advances in flexible electronic technology.^[^ ^9^, ^10^, ^11^, ^12^ ^]^ This technology has led to the development of wearable or skin‐attachable electronic sensors that can softly laminate onto the epidermis of a person, thus opened opportunities for the seamless and continuous assessments of the physiological status of humans, such as vital signs,^[^ ^12^, ^13^, ^14^ ^]^ body kinematics,^[^ ^15^, ^16^, ^17^, ^18^, ^19^, ^20^ ^]^ and chemicals in sweat.^[^ ^21^, ^22^, ^23^, ^24^ ^]^ Despite the rapid development of flexible wearable sensors for the human being, the development of plant wearable sensors is significantly left behind. Plant surfaces display far more diverse microscale features than the human epidermis, which are critical biointerfaces responsible for substances and energy exchange between plants and nature. Conventional man‐made sensing devices tend to perturb air/light/water/nutrient exchange at biointerfaces and their influences to plant long‐term viability remain mostly unknown. Therefore, despite a few successful examples of monitoring external characteristics such as the temperature and humidity on leaf surface, growth of plant organs (leaf, stem, fruit, etc.),^[^ ^25^, ^26^, ^27^, ^28^ ^]^ plant‐wearable sensor capable of noninvasive monitoring the internal physiological characteristics for individual plants have not been reported yet.

Herein, we present the first flexible electronic sensor that can harmlessly cohabitate with the plant and continuously tracking the sap flow in the plant, which is one of the most critical physiological characteristics for a plant, indicating the plant health, growth status, water consumption, and nutrients distribution and transportation.^[^ ^9^, ^29^ ^]^ By taking advantage of the recent advances in materials, micromechanics, and nanofabrication, our sensor is ultrathin, soft, stretchable, and lightweight, therefore can comfortably attach to the various plant surface (e.g., leaf and stem). The sensor also exhibits good physical stabilities, excellent water/air/light‐permeable, and biocompatibility. Therefore, the proposed sensor can live with plant surfaces and continuously monitor plant sap flow in the complex environment, e.g., agricultural farm field or greenhouse. Furthermore, a wireless communication unit and a customized software on a smartphone realized remote control and real‐time data acquisitionfor our sensor. We believe that our sensor holds great potential in studying individual plant biology, serving as a low‐cost, high‐throughput toolbox. Furthermore, the real‐time investigation on stem flow insides watermelon revealed an interesting day/night shift pattern of water allocation between fruit and its adjacent branch, which was previously unknown.

## 2 Results

### 2.1 Design and Fabrication of the Sensor

**Figure 1:**
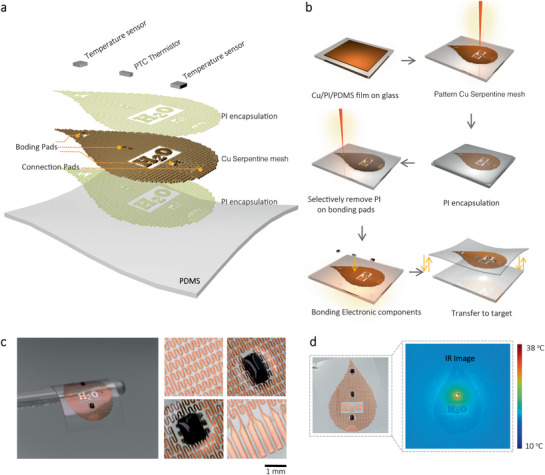
Design of the plant‐wearable sensor for sap flow analysis. a) Exploded view illustration of the device. b) Fabrication approaches of the plant‐wearable sap flow sensor. c) Optical images of the device, with enlarged images showing the serpentine interconnects and on‐boarded temperature sensor and thermistor. d) IR thermography with color and contrast enhancement highlights the spatial distribution of temperature when the thermistor operates for 120 s.

In this work, monitoring the sap flow is based on measurements of spatial anisotropy in thermal transport along the plant stem resulted from the flow. **Figure** **1**a presents the schematic view of the proposed sap flow sensor. The primary sensing components are two aligned temperature sensors with a positive temperature coefficient (PTC) thermistor in the middle. The operation of the thermistor will cause a sharp rise in local temperature. When there is a sap flow in the stem, since thermal transport is more efficient along the flow direction, an anisotropic temperature distribution occurs, which can be monitored by the two temperature sensors, thereby allowing the analysis of the sap flow rate. The abovementioned sensing components are binding to a serpentine copper (Cu, ≈6 µm) conductive tracks laminated between two polyimide (PI, ≈1 µm) layers to protect the flow sensor working in a harsh environment. The bottom layer is a thin (10 µm), stretchable, and air‐permeable polydimethylsiloxane (PDMS) film, providing physical support yet enabling robust, reversible adhesion to the surface of a plant. The whole multilayer structure with serpentine Cu tracks is very thin (≈20 µm), thus provides an extremely low effective elastic moduli, large deformability, and stretchability, allowing the sap flow sensor to adapt to the contour surfaces and time‐variate growth of the plant.

Figure 1b depicts the fabrication process of the sap flow sensor. Details of the fabrication approaches are described in the Supporting Information (Figure S1, Supporting Information). First, a thin Cu film (6 µm) supported by a glass slide was pattern into the serpentine tracks via a direct laser cutting technique. A thin PI layer (1 µm) was then spin‐coated on top of it to encapsulate the whole tracks. Then, the electronic chips were then bonded to Cu pads to form the flow sensing layer, after selectively removing the PI on the Cu pads by another laser cutting. Finally, the device was secured with an additional thin PDMS layer (≈10 µm) and was ready to pick up by a water‐soluble tape and transfer to the desired target. The above fabrication based on direct laser cutting technique is more efficient and faster than the conventional photolithography approach in preparing flexible electronic devices. Figure 1c illustrates the fabricated flow sensor supported and the detailed Cu tracks and sensor chips. The flow sensor is soft, flexible, small, and lightweight of 240 mg (≈20 mg cm^–2^) only, well meet the requirements for plant wearable sensors. The flow sensor can be connected to a wireless control system via flexible printed circuit board (FPCB) cable, therefore enabling remote control and data acquisition (see Figure S2, Supporting Information). Figure 1d shows infrared (IR) thermographs of the sensor revealing a spatial temperature distribution after the operation of the PTC thermistor for 30 s, indicating the success of the fabrication approach. Further information about the sensing system's integration is provided in the Supporting Information (Figure S2, Supporting Information).

### 2.2 Quantitative Analysis of a Sap Flow

**Figure 2:**
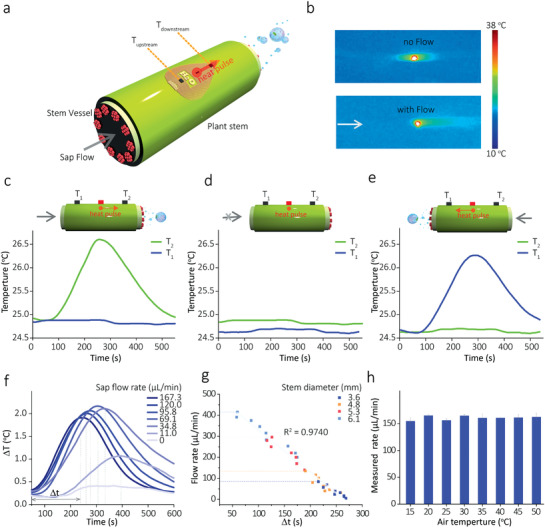
Detection principle of the sensing device. a) Schematic illustration of the detection principle of the sap flow sensor. b) IR thermography with color and contrast enhancement highlights the spatial distribution of temperature in the absence (top) and existence (bottom) of flow (120 µL min^–1^) when the thermistor is operating. Temperatures measured after the onset of heating for 120 s under three conditions: c) with a sap flow with direction left to right (120 µL min^–1^), d) absence of the flow, and e) with a reversed sap flow (120 µL min^–1^). f) Differential (Δ*T*) between the *T*_downstream_ and *T*_upstream_ responded to various flow rates. g) A liner fit for the flow rate (*R*) as a function of time (Δ*t*) is needed to reach the maximum Δ*T* after the heating. h) Responses of the flow sensor under different room temperatures.

**Figure** **2**a presents the illustration of the sensing mechanism of the sensor. Our sensor is mounted on a plant's stem. The operation of the PTC thermistor causes a sharp rise in local temperature. When there is a sap flow, since the heat will be transported along the flow direction, a pronounced anisotropy in the temperature distribution is created. Figure 2b, the IR thermography with color and contrast enhancement, indicates the anisotropy distribution of temperature in the presence of the flow. In contrast, temperature distribution around the PTC thermistor is almost symmetrical when there is no flow. With the two temperature sensors arranged in the flow direction on both sides of the PTC thermistor, then the anisotropic temperature distribution can be measured by the temperature sensors.

To verify the above assumption, the flexible sap flow sensor was mounted on a freshly cut watermelon stem, which was connected to a pump. By controlling the pressure of the pump, the rate and direction of the sap flow inside the stem can be adjusted. Details of the experimental setup were described in Supporting Information (Figure S10, Supporting Information). As shown in Figure 2c, the changes in local temperatures measured were recorded. When the flow was from left to right, the downstream sensor (*T*_2_) rose relatively faster than the upstream sensor (*T*_1_). In the absence of flow (Figure 2d), the heat transported equally along the stem, resulting in almost identical temperature values for *T*_2_ and *T*_1_. When the flow direction is reversed (Figure 2e), an expected opposite increase in temperatures is observed. The fluid transported heat from the thermistor to the *T*_1_, resulting in a higher and faster rise in *T*_1_ than *T*_2_. Therefore, the differential Δ*T* between the two sensors can give us an insight into the direction and rate of sap flow. Figure 2f shows the corresponding Δ*T* under different flow rates. We noticed that the observed maximal Δ*T* increased with the increase of the flow rate at the beginning. When the flow rate exceeded 120 µL min^–1^, the Δ*T* began to rise, due to the dominant convective effects of the flow leads to a cooling effect on both temperature sensors.^[^ ^30^, ^31^ ^]^ We also noticed that the time (Δ*t*) to reach the maximum Δ*T* at different flow rates is closely related to the sap flow rate.

Figure 2g indicates that a linear relation between the Δ*t* and the sap flow rate (*R*) was obtained. To understand the effect of stem size on the sensor's performance, we also used various stems (diameters 3.6–6.1 mm) to test the sensor. As shown in Figure 2g (also see Figure S11, Supporting Information), the obtained data can be well fitted by the same working curve with excellent linearity (*R* ^2^ = 9740). These results indicate our sensor's excellent adeptness, which can operate on the stem with different sizes. The lowest flow rate that can be determined by our sensor was 5 µL min^–1^. The operating range of our sensor was between 5 and 415 µL min^–1^. At the moment, no published data are available concerning the typical stem flow rates of watermelon. But judging from the sap flow range of a similar species greenhouse melon (*Cucumis melo* L.),^[^ ^32^ ^]^ our sensor should be operating in the range of watermelon.

Furthermore, we also investigated the influences of air temperature on the sensor. Figure 2h showed the measured rates between 15 and 50 °C (typical air temperature range in the farm) did not significantly differ (using ANOVA, *N* = 32, *p* = 0.079). The relative standard deviation of the measured rates was 5.66%. These results demonstrated the capability of our sensor in the determination of the stem sap flow rate.

### 2.3 Compliant of the Sensor

**Figure 3:**
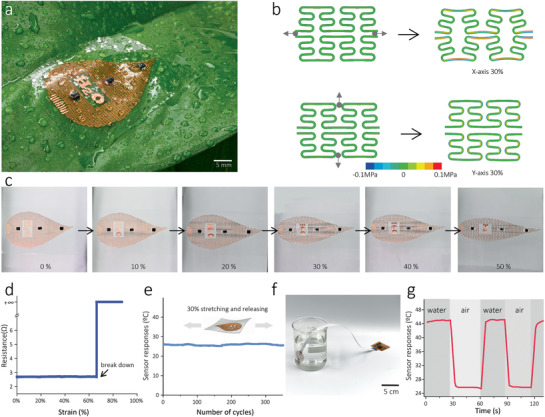
Mechanical characteristics of the device. a) Optical images of the flexible sap flow sensor mounted on a single plant leaf. b) Finite element analysis of the Cu serpentine mesh under mechanical stretching (30%) along the *x* and *y* directions. c) Optical images of the sensor device under five levels of uniaxial deformation up to 50%, showing no apparent structural fail in the mesh. d) Experimental result of the electrical resistance of the Cu mesh changing with uniaxial strains. e) Responses of the temperature sensor after repeated starching releasing. f) Optical image of a sensor wholly submerged in hot water (45 ℃), and g) the resulting temperature variation, indicating the excellent stability against the harsh environment.

The elasticity, mechanical robustness of the flow sensor under various deformations, and harsh working environments like agricultural fields are essential requirements for plant wearable sensor systems. **Figure** **3**a shows an optical image of the sensor mounted on a pathos leaf. It indicates that our sensor is light enough (240 mg) for a small plant's leaf to bear, thus suitable for plant‐wearable sensing applications. Ultrathin design and serpentine conductive tracks were used to increase the tolerance to mechanical deformations and prevent the electronic circuit's mechanical fracture. According to the previous reports,^[^ ^33^, ^34^, ^35^ ^]^ this fractal layout can maximize stretchability along the stem axes. The mathematical model of the serpentine design can be found in this reference.^[^ ^36^ ^]^ Meanwhile, the finite element analysis simulation provides a versatile tool for analyzing the response of the fractal layout to applied strains. As shown in Figure 3b, the serpentine Cu tracks showed excellent flexibility, which can accommodate sizeable uniaxial stretching (also see Figure S3, Supporting Information). Despite that our flow sensor contains chip‐scale components, the whole system can stand a large deformation (50%) without breaking (Figure 3c), due to the adoption of a “island‐bridge” strategy^[^ ^12^, ^34^ ^]^ that can isolate deformations to the soft elastomer and alleviate stress concentration on the chip (also see Figure S4, Supporting Information). Figure 2d revealed that the Cu tracks' resistance remained relatively stable (±0.01 Ω) below the rapture point. This small variation will not affect the sensor's performance, especially considering that our sensor relies on digital signals, not analog. These results indicate that the flexible sap flow sensor is sufficiently stretchable and stable that allows accommodating large deformation on complex biointerfaces.

The durability of the flow sensor was also investigated. As shown in Figure 3e, we noticed the flow sensor was working normally even after 350 cycles of a tensile strain (30%), translating into a 9 mm elongation. The flow sensor also exhibited excellent waterproof quality. We noticed that the temperature sensor was functioning normally even after wholly submerged in water, as shown in Figure 3f,g. Such features allow the flow sensor to operate appropriately in a hazardous environment. These results indicated our sensor's good mechanical robustness, which can perfectly fulfill the requirements of a reliable and long‐term plant wearable sensor.

### 2.4 Biocompatibility of the Plant‐Wearable Sensor

**Figure 4:**
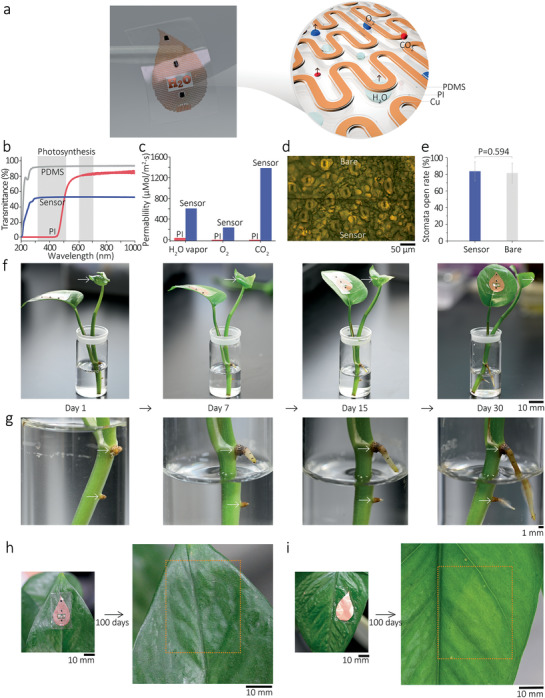
Biocompatibility of the plant‐wearable sensor. a) Schematic illustration of the interstice design of the serpentine traces to achieve gases/light‐permeable and waterproof properties for the sensor. b) Light transmittance spectra of our sensor, pure PDMS thin film (20 µm), and PI film (30 µm). Shadow areas indicate the wavelength range of the light essential for photosynthesis (315–520 and 600–700 nm). c) Comparison of H_2_O vapor, O_2_, and CO_2_ permeability for our sensor and PI film (10 µm). d) Micrograph of the stomata on a leaf in sensor mounting area and unmounted range. e) Comparison of the stomata open rates on the leaf in the sensor mounting area and the unmounted area was obtained in 40 randomly chosen areas (≈500 × 500 µm). No statistically difference was overserved (*p* = 0.594, *N* = 40). f) Optical images of a sensor‐mounted pothos seedling during 30 days of indoor growth in culture solution. Note the seedling exhibited clear signs of health and vitality, and g) intensive growth of roots was observed. h) Optical images of the sensor‐mounted area on a pothos leaf after 100 days of indoor growth in soil under moderate sunlight. Note the site maintained healthy green in color, indicating chlorophyll production was normal. However, as a control test, i) optical images of a PI film‐mounted area (resembling the traditional type sensor) show the mounted area turned yellow, indicating a decrease in chlorophyll content.

Plants surfaces are critical biointerfaces responsible for substances and energy exchange between plants and the environment. Conventional electronic sensing devices tend to block air/light/water/nutrient transport and perturb the plant's long‐term viability. However, due to the special design of our sensor and the materials chosen (shown in **Figure** **4**a), light and gases allow passing through the interstices of the narrow Cu meshes. Figure 4b shows the light transmittance spectra of our sensor, revealing a good optical transmittance of our sensor across the UV to the near‐infrared region (≈300–1000 nm). In contrast, the PI film (resembling the traditional type rigid sensor) showed low light transmittance between the wavelength of 315–520 nm (blue light), one of the essential ranges for chlorophyll's photosynthesis. Meanwhile, some gases (such as H_2_O vapor, O_2_, and CO_2_) allow passing through the interstices of Cu meshes and the gas‐permeable PDMS supporter of our sensor (see Figure 4c; Table S3, Supporting Information). These gases are critical to photosynthesis and transpiration processes. In contrast, the PI film showed very poor gas permeabilities of H_2_O vapor, O_2_, and CO_2_. Furthermore, to study the cytotoxicity of the sensor, NIH/3T3 fibroblasts were used as the model cell. As shown by the results in Figure S7, Supporting Information, the expression of live cells in the sensor (Figure S7d, Supporting Information) was similar to that in the negative controls (Figure S7a,b, Supporting Information), and dead cells were rarely observed. In contrast, almost all cells in the PI/Cu group (Figure S7c, Supporting Information) were dead. These results suggest the excellent biocompatibility of the sensor.

To understand the long‐term influence of our sensor to plant's viability, we mounted the sensor on the leaf of a small pothos seedling and investigated their growth characteristics over time. Since leaf is the primary tissue for photosynthesis, respiration, and transpiration, the influence of sensor on leaf growth (if there is) would be easier to observe than on other tissues such as stem. Thus, we chose leaf instead of stem for this study. After deploying the sensor, as shown in Figure 4d,e, no significant difference was observed in the stomata open rates between the sensor mounting area and unmounting area (*p* = 0.594, *N* = 40), indicating our sensor would not perturb the respiration and transpiration of the plant leaves. After 30 days of indoor growth in water and under moderate sunlight, the seedling displayed clear signs of health and vitality (Figure 4f). A new leaf began to grow in the seedling (Figure 4f), and intensive root growth was observed in the vase (Figure 4g). After 100 days, as shown in Figure 4h, the leaf contained the sensor maintained healthy green in color, indicating that the production of chlorophyll in the mounted area is unaffected (also see Figures S8 and S9, Supporting Information). On the contrary, the PI/Cu mounted area turned yellow, indicating a decrease in chlorophyll content (Figure 4i). All the evidence suggested that our plant wearable sensor exhibited excellent biocompatibility and can harmlessly live with the plant for a relatively long time.

### 2.5 Sap Flow Monitoring in Fields

**Figure 5:**
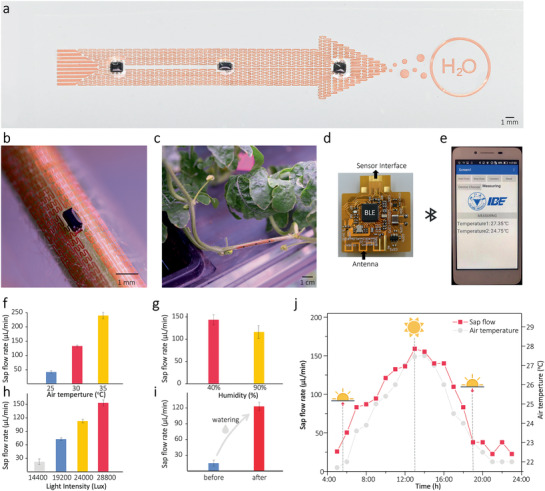
Real‐time monitor fluctuation of sap flow in watermelon in the real farm field. a) Optical image of an “arrow”‐shaped sensor. b,c) Optical images of a healthy watermelon plant with a sensor mounted on its stem. d) Images of the wireless control system and e) a custom‐developed smartphone application that enables remote control, wireless data acquisition and graphics display of the sensor.The measured sap flow rate under various f) temperature (25, 30, and 35 ℃), g) relative humidity (40% and 90%), and h) illumination intensity (9600–28 800 lux). i) The variation of sap flow rates of the watermelon plant after watering. j) Continuously monitoring the watermelon plant's sap flow rates in the field for 18 h (05:00–23:00).

To demonstrate the capability of the flow sensor in the real farm field, watermelon was chosen as a model due to its relatively strong stem sap flow caused by large water consumption during the maturity stage. For the convenience of deploying the sensor on the stem, an “arrow”‐shaped sap flow sensor was developed, as shown in **Figure** **5**a. The basic layout of the sensor is the same as the flow sensor mentioned above, including the relative positions of the heater to temperature sensors, and the fractal structure of the Cu traces. Watermelon stems with a diameter of ≈5 mm and a typical hairy surface (1–2 mm long) were used in this experiment. The results showed in Figure 5b,c indicated our flow sensor could conformally and firmly attach to the stem's surface despite the soft hairs on the stem (also see Figure S13, Supporting Information). A wireless control system that enables remote control and data acquisition was developed (Figure 5d), which can be connected to the sensor via flat FPCB cable (see Table S1, Supporting Information, for the technical specification). Figure 5e shows a custom‐developed smartphone application that enables remote control of the sensor (on/off), wireless data acquisition, and graphics display simultaneously. The total weight of the communication system and the flow sensor is about 4 g, light enough for a single plant stem to bear (see Figure S2a,b, Supporting Information).

In situ measurements of the stem sap flow were conducted. Figure 5f–i shows the plant's sap flow responses to various environmental stresses, such as temperature, humidity, solar radiation intensity, and soil moisture (also see Section S6.1, Supporting Information). As shown in Figure 5f, rise in the temperature significantly increased the sap flow rate due to the increase in the plant's transpiring activities. On the contrary, rise in humidity level significantly decreased water consumption in the plant transpiration, therefore reducing the sap flow rate (Figure 5g). The transpiration rate of the plant was closely related to light intensity. Stronger light increases the water need for photosynthesis and perspiration, as well as the sap flow rate (Figure 5h). Besides, soil moisture acts as the “source” for the sap flow activities. As shown in Figure 5i, we noticed the stem sap flow increased significantly after watering.

Continuous measuring on the watermelon stem sap flow was conducted in a farm field for 18 h, sufficient time to see the sap flow fluctuation. As shown in Figure 5j, the sap flow rate increased significantly after sunrise (05:00–13:00) with the rising temperature. Between 13:00–14:00, the flow rate began to decrease with continuously rising temperatures, which might relate to the stomatal closure, a defense mechanism of plants against high temperatures and/or high‐level solar radiation. After that (14:00–23:00), the sap flow rate continued to decrease with the air temperature and solar radiation fall. These phenomena were consistent with the previous reports that measurements on large plants (such as trees) using invasive or destructive approaches,^[^ ^29^, ^30^, ^37^, ^38^, ^39^ ^]^ however, can be successfully observed in the small herbaceous plant in a continuous and non‐destructive manner using our sensor.

### 2.6 In Situ Monitoring of Water Distribution inside a Plant

**Figure 6:**
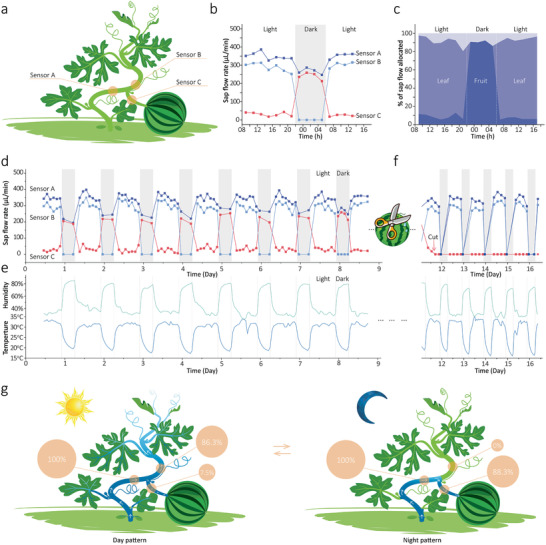
In situ monitor of the water allocation inside watermelon plant. a) Schematic illustration of the sensors' deployment (sensor A: basal stem; sensor B: leaf branch; sensor C: fruit branch). The watermelon plant was cultivated in a phytotron (06:00–22:00 light conditions at 30 °C and 22:00–06:00 dark conditions at 20 °C). b) Typical variation of the stem sap flow rates measured at these positions, and c) the corresponding calculated water allocation between the fruit and leaf branches. Continuous measurement of the stem sap flow rates d) before and f) after the watermelon fruit was harvested. e) The corresponding temperature and humidity in the greenhouse during the measurements. g) Schematic illustration of day and night patterns of thesap flow in watermelon. The water allocation rates were averaged from the corresponding periods in the entire 16 days of measurements.

To investigate the water allocation inside the watermelon, several sensors were mounted on the basal stem and two adjacent branches near a watermelon fruit, which were grown in a phytotron (16 h light conditions at 30 °C and 8 h dark conditions at 20 °C). As shown in **Figure** **6**a, sensor A was monitoring the flow in the basal stem, while sensors B and C measured the flows in the leaf branch (apical branch without any fruit) and fruit branch simultaneously (also see Figure S14, Supporting Information). Figure 6b,c shows the variations of flow rates at these positions in a typical day and the calculated water allocation rates between the two branches accordingly. We noticed that during the light period (06:00–22:00), the majority of water (86.3%) from the basal stem was allocated to the leaf branch, due to a high level of photosynthesis and transpiration on the leaves. In contrast, only 7.5% of the water flowed to the watermelon fruit. During the dark time (22:00–06:00), the water flow to the leaf branch stopped (sensor B) with the cease of photosynthesis and perspiration activities, and the water in the basal stem also decreased a little (sensor A). Surprisingly, we noticed that the water flow into the fruit branch dramatically increased nearly ten times (from 25.7 to 228 µL min^–1^). Almost all (88.3%) of the water in the basal stem was allocated to the watermelon fruit. Noticeably, Figure 6c also indicated a small amount of water loss from the basal stem (the gray area), which could be consumed by the plant tissues between the sensors or measurement errors.

The light/dark shift pattern of water allocation was stably observed in the consecutive 12 days (Figure 6d). Figure 6e also provided room temperatures and humidity in the greenhouse. Accordingly, we found the observed fluctuations of sap flow rates in Figure 6d are highly consistent with the previously discovered influences of temperature and humidity on the flow rates (shown in in Figure 5f,g), indicating the reliability of our measurements.

We also noticed the flow to fruit (Figure 6f, sensor C) immediately turned to zero after the fruit was harvested. Meanwhile, all the night's flows (including the basal stem) completely stopped, implying that the observed night's flow in the basal stem before the fruit harvesting (Figure 6b,c, sensor C) was entirely driven by the watermelon fruit. Figure 6g schematically illustrates the light/dark shift patterns of watermelon's water allocation and the main driving forces for stem sap flow. During the daytime, the main force is the photosynthesis and transpiration in the leaves, while at night, the imbibition of expanding watermelon fruit (fruit growth) might be the key contributor. These findings imply that the accumulation of fruit fresh weight mainly happens during the night, which is contradicted with the common view of believing the fruit growth rate should be synchronized with the photosynthesis activities, in other words, higher during the daytime. To the best of our knowledge, this phenomenon has not been published before.

## 3 Discussion

Sap flow symbolizes the translocation of plant food, including water, nutrition, and photosynthetic product, which contributes largely to the growth of plant shoot parts.^[^ ^9^, ^39^ ^]^ Thus, the monitoring of sap flow would provide clues about the plant status. Furthermore, the sap flow provides a unique window to study the plant behaviors to various environmental stresses (e.g., water, temperature, humidity, and light), as well as for phenotyping (e.g., the selection of drought/heat tolerant species). Compared to conventional phenotyping approaches, the responses of sap flow to these stresses come much faster (as shown in Figure 5f–i) than the consequences observed in the plant morphologies (such as plant height, leaf color, fruit size, etc.). Therefore, phenotyping efficiency could be significantly improved.

To quantitatively understand the effects of water change on plant physiology and ecological processes, technologies for measuring sap flow rate are highly desirable. The methods commonly used nowadays include dry/weighing methods, traditional invasive probe sensors, etc.^[^ ^38^ ^]^ Such methods are complicated and time‐consuming or destructive; thus, continuous monitoring of the sap flow for a relatively long period for plants is not possible.^[^ ^29^, ^39^ ^]^ The recently reported flexible/wearable electronic flow sensors, based on analyzing the time/spatial thermal responses of tissues to the fluids, shed light on how to non‐destructively measure flows in complex biointerfaces, such as blood flow,^[^ ^40^, ^41^, ^42^ ^]^ cerebrospinal fluid,^[^ ^43^ ^]^ sweat and interstitial fluid,^[^ ^44^ ^]^ and airflow.^[^ ^45^, ^46^, ^47^ ^]^ However, a plant‐wearable sensor that can continuously monitor its sap flow and harmlessly cohabitate with a plant is still challenging. The first challenge is to fabricate a sensor light in weight, sufficiently flexible and stretchable, and to anchor any desired position of plants for sap flow measurement. To meet this challenge, we developed a fabrication approach based on direct laser cutting technology, which is far more efficient than the conventional photolithography approach in preparing this type of flexible electronics. More importantly, due to our sensor's unique design and the chosen materials, the obtained sensor is thin, air/water/light‐permeable, and biocompatible, resulting in few damages to plant and no obstacle to their growth and normal physiological processes.

Our sensor is practically suitable for the rapid detection of the sap flow rate for plant phenotyping. Our sensor's key advantage over the traditional plant sap flow sensors is the wireless data acquisition capability. Furthermore, since we only chose commercially available low‐cost chips and materials for the sensor, each sensor's fabrication cost may be expected to be less than 10 US dollars (see Table S2, Supporting Information). Consequently, the requirement of low‐cost and reliability for plant phenotyping can also be met. A future work should be dedicated to an automictic phenotyping platform capable of simultaneously monitoring thousands (or more) of targets in a highly controlled environment, enabling high‐throughput measurement, minimizing the influences of environmental effects, and revealing the true biological causes of the genes. With this platform, it should be possible to boost the plant phenotyping to the next level.

One limitation of our sensor is it is not suitable for large plants with thick barks (such as trees), which are often measured by commercialized sensors using invasive sensing probes. However, our sensor may bridge the gap by providing a tool for sap flow measurement in most herbaceous crops, which could not be examined via invasive sensors. Further application of our sensor to other plant species or organs should be carefully evaluated, since the diversities in the plant's structures and thermal transport properties could lead to unsuccessful measurement. For example, we also tried but failed to measure the flow in leaf, because the layout of leaf veins is significantly different from the stem. For long‐term studies, the influence of plant growth (e.g., extension and thickening of the stem) on the sensor performance should be considered, especially for the plants that have intercalary meristem (such as wheat and corns). In this work, the extension of the stem can be neglected (less than 1%, shown in Figure S15, Supporting Information) because the growth of the watermelon stem always derives from cells at the tip of the stem (shoot apical meristem, SAM) while the length of the grown stem remains unchanged.

In conclusion, we extended recent epidermal electronic devices to generate a soft, plant‐wearable sensor that can accurately measure the sap flow rate in a continuous, noninvasive, and real‐time manner. This work presents the first flexible electronic sensing device that can harmlessly cohabitate with plant and continuously monitor its sap flow. The potential applications of our sensing devices are vast, ranging from monitoring the health status of the plant to agricultural production. Especially, our sensor holds great potential in plant phenotyping, serving as a noninvasive, high‐throughput, and low‐cost toolbox.

## 4 Experimental Section

##### Fabrication of the Plant‐Wearable Sap Flow Sensor

A Cu film (6 µm thickness) was first laminated by a PI layer (1 µm) by spin coating. The Cu film was then fixed onto a glass slide coated with a cured PDMS layer (10 µm) as a temporary adhesive layer, followed by defining a Cu serpentine mesh by laser cutting (LPKF U4 laser system, LPKF Laser & Electronics AG, Germany). Then, a 1 µm PI layer was spin‐coated on the sensor. A second laser cutting was applied to remove the PI for exposing the connection pads for the chip components, and the PTC thermistor and temperature sensors were welded on the pads with the solder pasting. At last, to make the sensor waterproof, the whole sensor was packaged using a additional thin layer of PDMS (≈10 µm). A wireless electronic instrumentation circuit was developed to control the thermistor, obtaining the temperature information, and transferring it to a PC or smartphone (see the Supporting Information).

##### Calibration of the Sensor and In‐Field Monitoring of a Sap Flow Rate

An experimental setup for calibration of the sensor is described in the Supporting Information (Figure S10, Supporting Information). In brief, a sap flow sensor was mounted in the middle of a fresh‐cut watermelon stem (length 15 mm, diameter ≈4 mm) that was connected to a peristaltic pump with a stainless steel ferrule and a rubber tube. By adjusting the pump pressures, various sap flow rates could be obtained, and the responses of the sensor were recorded accordingly. The exact water flow rates were determined by weighting water flow through the stem in a certain period. A linear relation between the sap flow rate (*R*) and Δt was found.

Real‐time determination of the sap flow rate in the field was conducted on a healthy watermelon plant in the maturation stage. Details can be found in the Supporting Information (Figure S11, Supporting Information). The plant was grown in a farm field under ambient conditions. A sensor was mounted on a root stem with a diameter of ≈4 mm. To see the sap flow fluctuation, the monitoring was done continuously for 18 h (between 05:00 and 23:00). For each measurement, the thermistor heated for a short period (120 s), and the resulting temperature variations downstream and upstream were recorded during the following 360 s. A total of ≈5 min was required for each measurement.

##### Finite Element Analysis of the Sensor

3D finite element analysis allowed predicting the mechanical deformations and the Mises stress distributions of the network designs. The material parameters used are *E*_PDMS_ = 1.8 MPa, *ν*_PDMS_ = 0.48 for PDMS; *E*_PI_ = 4.5 GPa, *ν*_PI_ = 0.34 for PI; and *E*_Cu_ = 119 GPa, *ν*_Cu_ = 0.326 for Cu. Here, *E* is the elastic modulus and *ν* is the Poisson's ratio. Refined meshes ensured computational accuracy.

##### Instruments

IR thermography was collected using an infrared camera (Fluke, Ti110) and analyzed with SmartView 2017 Software. The resistance of the sensor was measured by Digit Multimeter (Keithley, DMM7510). Strain and starch analysis of the device was performed by a universal testing machine (Sunstest, UTM2203). The optical transmittance spectra of the sensor were measured using a UV/vis spectrophotometer (Evolution 300, Thermos Fisher Scientific, USA) over the typical Cu serpentine traces area, as indicated in Figure 4a. Optical images were obtained using Nikon Z6 Camera.

## Conflict of Interest

The authors declare no conflict of interest.

## Supporting information

Supporting Information

## Data Availability

The authors confirm that the data supporting the findings of this study are available within the article and its supplementary materials.
