# Supplementary material for: Cohabiting Plant‐Wearable Sensor In Situ Monitors Water Transport in Plant
Source: Adv Sci (Weinh). 2021 Mar 9;8(10):2003642. doi: 10.1002/advs.202003642 (PMC8132156; doi:10.1002/advs.202003642)
Supplement: Supplementary file 1 — Supporting Information [file ADVS-8-2003642-s001.pdf]

© 2021 Wiley-VCH GmbH

ADVANCED SCIENCE
Open Access

Supporting Information

for *Adv. Sci.*, DOI: 10.1002/advs.202003642

Cohabiting Plant Wearable Sensor in-situ Monitors Water Transport in Plant

Yangfan Chai,[#,1] *Chuyi Chen,*[#,1] *Xuan Luo,*[1] *Shijie Zhan,*[2] *Jongmin Kim,*[2] *Jikui Luo,*[3] *Xiaozhi Wang,**[3] *Zhongyuan, Hu,*[*,4] *Yibin, Ying,* [*,1] *Xiangjiang Liu*[*,1]

Supporting Information

Cohabiting Plant Wearable Sensor in-situ Monitors Water Transport in Plant

Yangfan Chai, [#,1] *Chuyi Chen,* [#,1] *Xuan Luo,* [1] *Shijie Zhan,* [2] *Jongmin Kim,* [2] *Jikui Luo,* [3] *Xiaozhi Wang,* [*,3] *Zhongyuan, Hu,* [*,4] *Yibin, Ying,* [*,1] *Xiangjiang Liu* [*,1]

[1.] College of Biosystems Engineering and Food Science, Zhejiang University, Hangzhou, 310058, China

[2.] Department of Engineering, University of Cambridge, Cambridge, CB3 0FF, UK

[3.] College of Information Science and Electronic Engineering, Zhejiang University, Hangzhou, 310058, China

[4.] College of Agriculture and Biotechnology, Zhejiang University, Hangzhou, 310058, China

[#] These authors contributed equally to this work.

* Corresponding Authors. Email: xjliu@zju.edu.cn, xw224@zju.edu.cn, huzhongyuan@zju.edu.cn, yingyb@zju.edu.cn

1. Materials

Cu film (thickness 6 μm, D-6, Jingliang Copper Co., Ltd, China)

Polyimide (YD4001, YiDun New Material Company, SuZhou, China)

PDMS (Sylgard 184, Dow Corning, China)

Anisotropic conductive film (CP37031YC, Hitachi Chemical Co., Japan)

Water-soluble adhesive tape (3M, Mouser Electronics, USA)

PTC Thermistor (PRF18BC471RB5RB, muRata, Japan)

Temperatures sensors (TMP102, Texas Instruments, USA)

Solder paste (SMDLTFP250T3, Chip Quik Inc, USA)

2. Design and Fabrication of the sensor

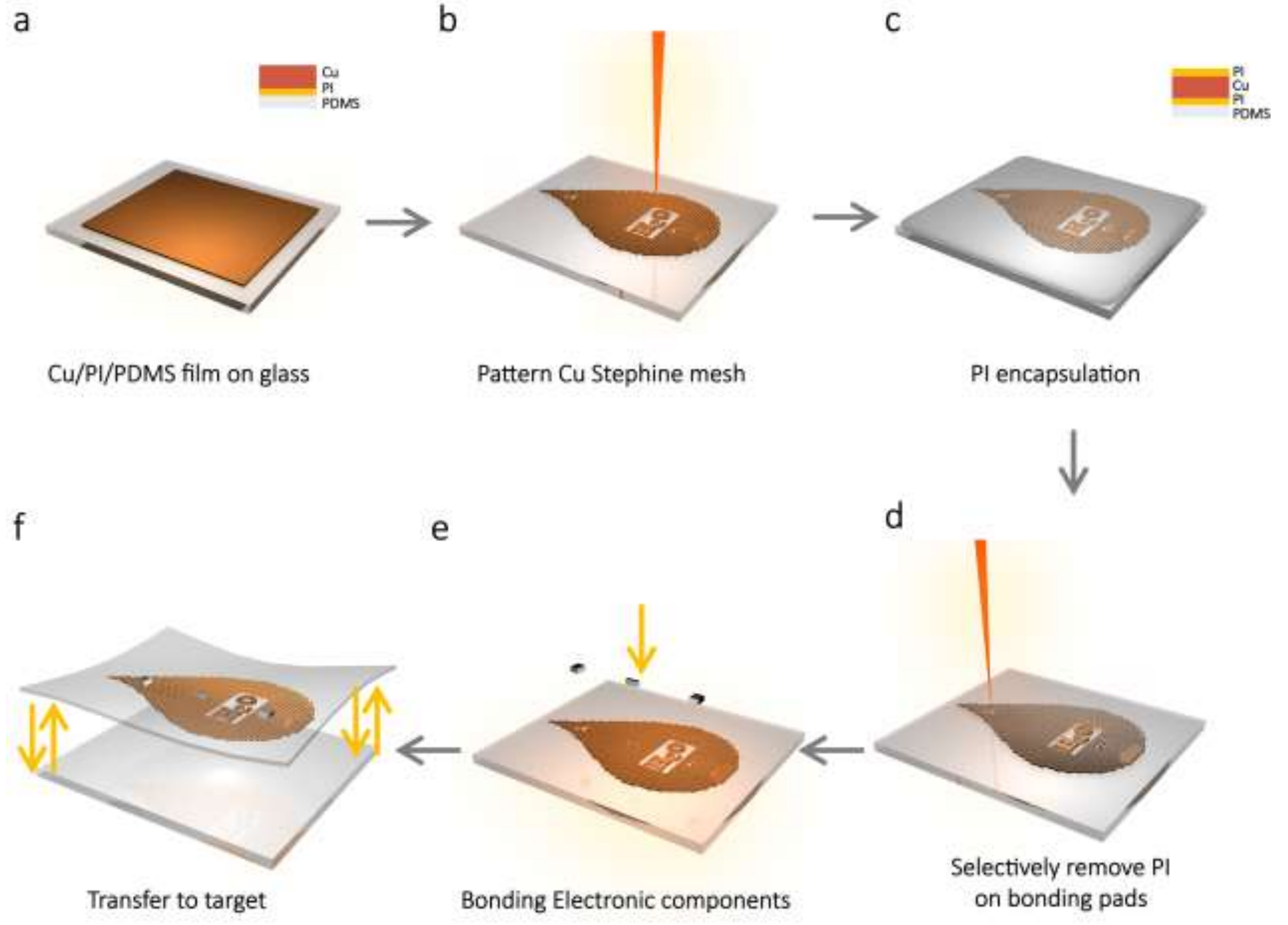

Figure S1. Process for fabricating the Sensor. (a) A Cu film (6 µm thickness) was firstly spin coating (3000 rpm, 45 s) with a layer of polyimide (2 µm) and curried at 200 °C for 2.5 h. Then the Cu film was fixed onto a glass slide coated with a layer of cured PDMS (10 µm) as a temporary adhesive layer, **(b)** followed by defining Cu serpentine mesh by laser cutting (LPKF U4 laser system, LPKF Laser & Electronics AG, Germany). **(c)** Then, a 2 µm PI layer was spin-coated (3000 rpm, 45 s) on the top of the mesh. **(d)** A second laser cutting was applied to remove the PI for exposing the connection pads for the chip components. **(e)** The thermistor and temperature sensors were welded on the pads with the solder pasting. **(f)** At last, the whole sensor was transferred with water-soluble tape and secured with a layer of PDMS. The sensor was then ready to connect to the control unit via anisotropic conductive film (ACF) tape.

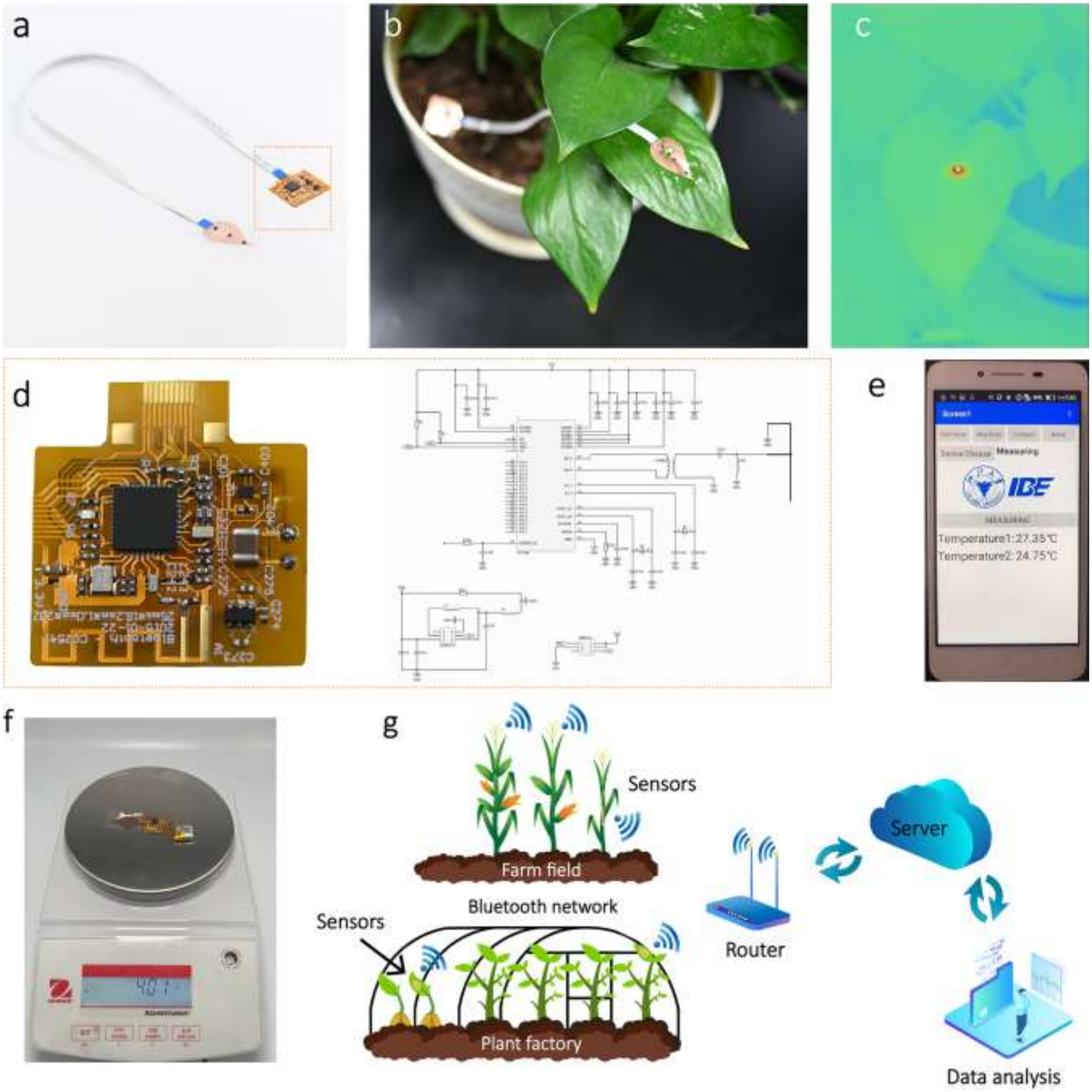

Figure S2 Wireless communication. (a) images of a Sap flow sensor connected with the wireless communication and control system via a flat FPCB cable. **(b)** Deployment of the sensing system on a leaf. **(c)** IR image of an operating sensor. **(d)** the wireless communication and control system and its schematic circuit diagram. The PCB was designed on a 150 µm polyimide flexible substrate. The core system uses a Texas Instrument CC2541 2.4-GHz BLE and Proprietary System-on-Chip device, which could be programmed on board by IAR Embedded Workbench for ARM. The microcontroller could

receive the data from the temperature sensor (TMP102, Texas Instrument) *via* the Inter-Integrated Circuit Bus (IIC) protocol and transfer it to a smartphone or PC via Bluetooth. The circuit was powered by a single rechargeable Li-ion polymer battery with a nominal voltage of 3.7V, stabilized by a DC-DC converter (TPS62332, Texas Instrument). A DC-DC converter (PS3120A, Pulan Technology) was used to power up the thermistor to boost the battery voltage from 3.7V to 5V. The time of heating could be customized by controlling the voltage of the pin from PS3120A. **(e)** A custom-developed Android application interface for control and data acquisition. **(f)** Total weight of the sensing system, including the sensor, control system, and the batterie. **(g)** The design of a proof-of-concept plant phenotyping platform allows multiple sap flow sensors to work simultaneously to realize high-throughput measurement. The platform is based on the wildly-used Bluetooth networking technology. The various plants' physiological information is gathered by the sensor array and sent to a router via Bluetooth protocol. The router would send the data to the Server or Cloud through the TCP/IP protocol. Then, the information along the respective genetic information can be automatically analyzed by a custom-developed software.

Table S1 Technical specification of the control system

PARAMETER	SPECIFICATION
**Size**	25 mm × 25 mm × 1 mm<br>(Long × Wide × thickness)
**Weight**	4 g (Sensor 0.24 mg; FPCB 0.8 g; Battery 3g)
**Wireless connection**	BLE 4.0
**Frequency**	2.4-GHz
**Main Chip**	CC2541
**Memory**	in-system 256 KB programmable flash memory, 8KB of RAM
**BLE Antenna**	Meandered inverted FPCB antenna
**Supply voltage**	3.7 V
**Battery Capacity**	200 mAH
**Compatible system**	Android 5.0 or higher
**Recommended Operating Conditions**	0 – 50 °C

Table S2 Estimation of Cost. The prices of the chips were obtained from the website (https://www.alldatasheet.com, accessed in June 2020).

**Chip**	**Price ($)**	**Quantity**	**Cost ($)**
PTC Thermistor (PRF18BC471RB5RB, muRata, Japan)	0.1921	1	0.1921
Temperatures sensors (TMP102, Texas Instruments)	0.5402	2	1.0804
Capacitor voltage doubler （C1210）	0.09746	23	2.24158
CC2541 (Texas Instruments)	1.864	1	1.864
TPS62730 (Texas Instruments)	0.4612	1	0.4612
PS3120A (Pulan Technology, China)	0.03	1	0.03
LFB182 (muRata, Japan)	0.131	1	0.131
BLE	0.51943	1	0.51943
Crystal oscillator（49SMD）	0.107	2	0.214
Resistance（0805）	0.02	10	0.2
li-ion battery (300mA)	1.21	1	1.21
**Total Cost**			**8.14371**

3. Characterization of the Sensor

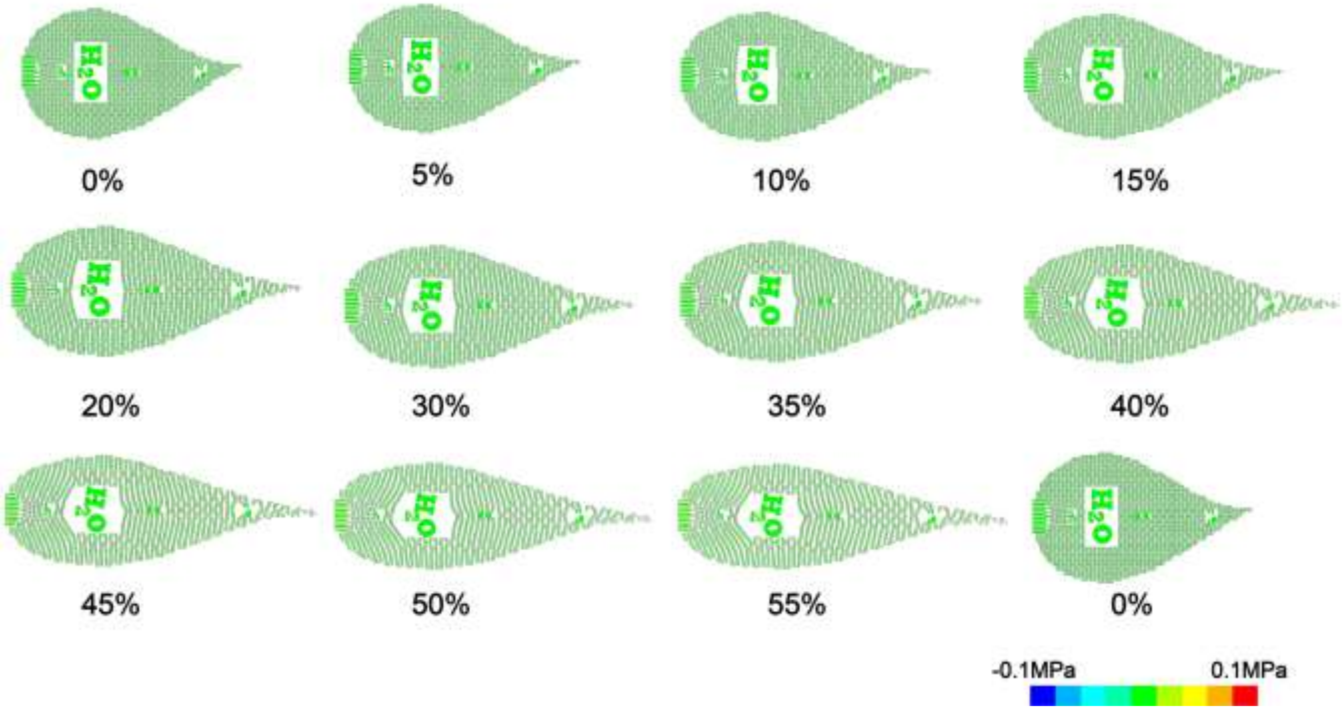

Figure S3 Finite element analysis of the mechanics of the sensor. System-level deformations under uniaxial stretching and Mises stress distribution determined by FEA of the sap flow sensor. The material parameters used are: E_{PDMS} = 1.8 MPa, ν_{PDMS} = 0.48 for PDMS; and E_{Cu} = 119 GPa, ν_{Cu} = 0.326 for gold. Here, E is elastic modulus, and ν is Poisson's ratio.

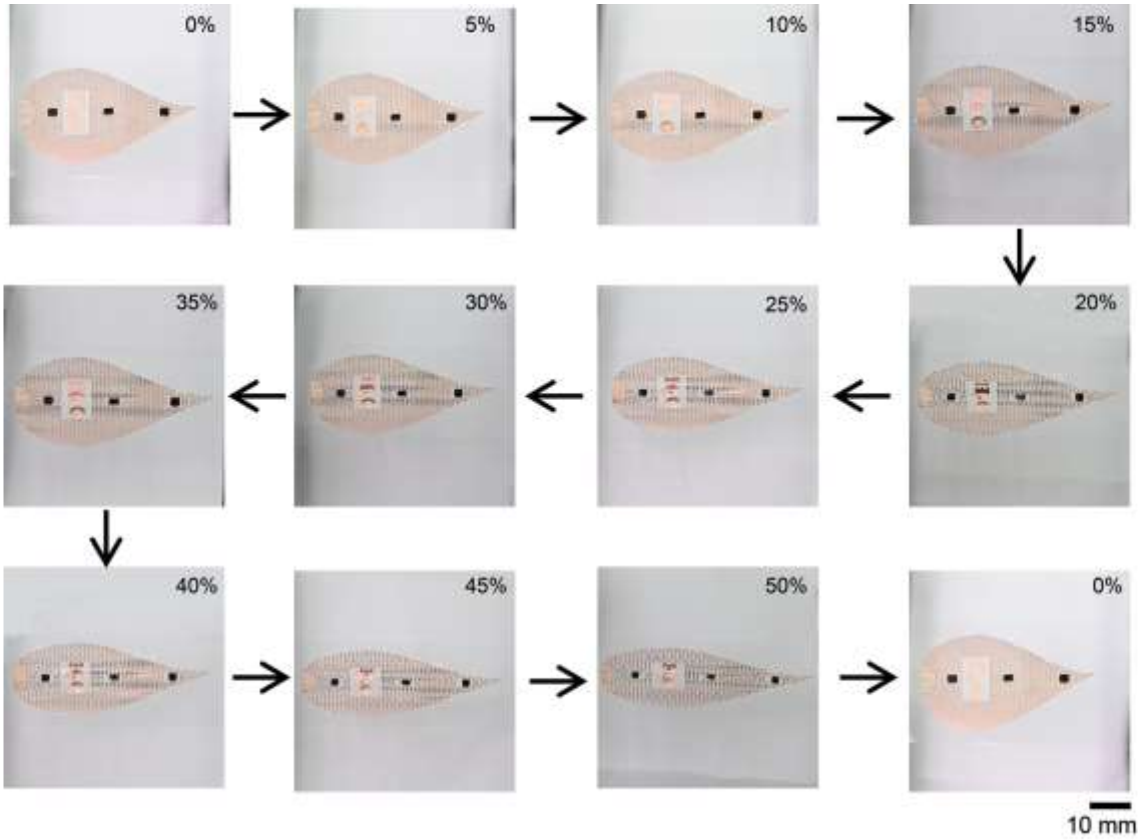

Figure S4 Optical images of the sensor under different strains (0 - 50%).

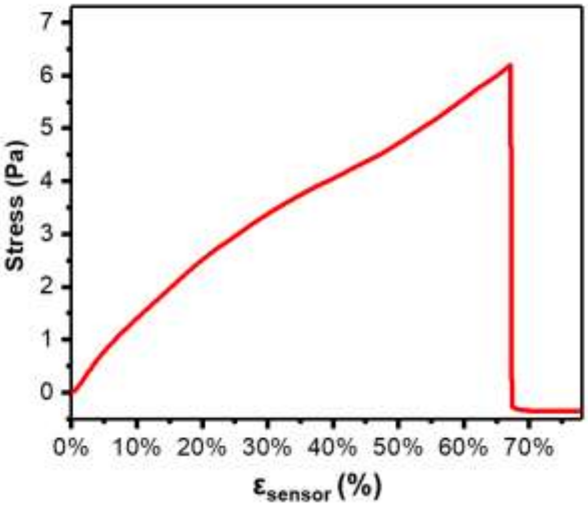

Figure S5 Stress-strain curve of the sap flow sensor.

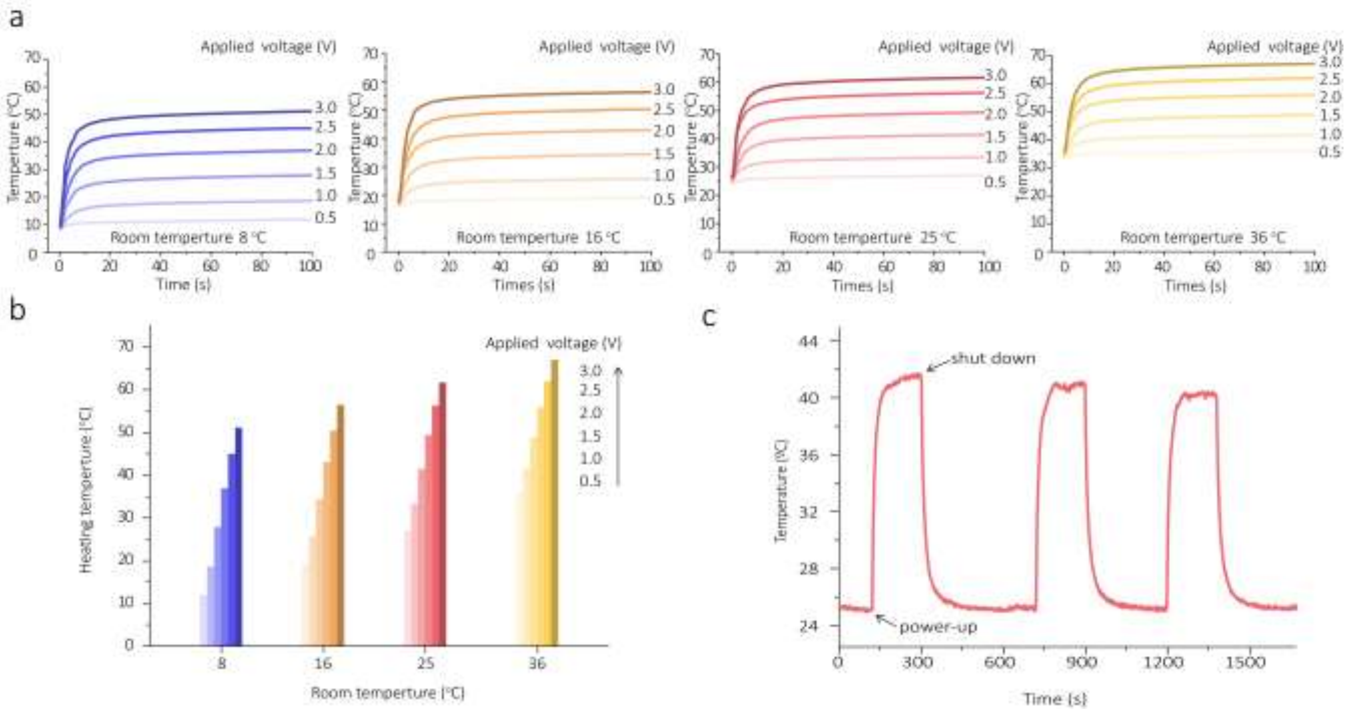

Figure S6 (a) Operation of the PTC thermistor and (b-c) the resulting heating temperatures. PTC thermistor is self-regulating heaters that run open-loop without additional regulating electronics or sensors. It can automatically reduce the power when the temperature increases and switch off the heat production above a specific temperature. PTC heaters typically keep a constant temperature within a reasonable tolerance, regardless of the ambient conditions. As a result, the heater inherently eliminates the risk of overheating.

4. Biocompatibility analysis of the sensor

Table S3 Gas permeability of different materials. Water vapor transmission rates were determined by weighting method according to the Japanese standard method (JIS K 7129-1:2006, measuring conditions: temperature 38 ^{o}C, relative humidity 90%, sample size 33 cm^2). O_2 and CO_2 transmission rates were determined by differential-pressure method according to Japanese standard (JIS K 7126-1:2006, measuring conditions: temperature 23 ^{o}C, relative humidity 0%, pressure 0.1MPa, sample size 4.9 cm^2).

	**H_2O vapor (g/m^2·24h)**	**O_2 (cm^3/m^2·24h)**	**CO_2 (cm^3/m^2·24h)**
**PI film (10 μm)**	56.6313	223.477	841.449
**Our sensor**	**940.0498**	**462064.36**	**2686836.83**
**PDMS film (20 μm)**	1138.6039	1027000.00	3504000.00

	**H_2O vapor (μMol·m^{-2}·s^{-1})**	**O_2 (μMol·m^{-2}·s^{-1})**	**CO_2 (μMol·m^{-2}·s^{-1})**
**PI film (10 μm)**	36.4141	0.115471	0.434776
**Our sensor**	**604.455**	**238.74853**	**1388.28745**
**PDMS film (20 μm)**	732.126	530.06501	1810.0516

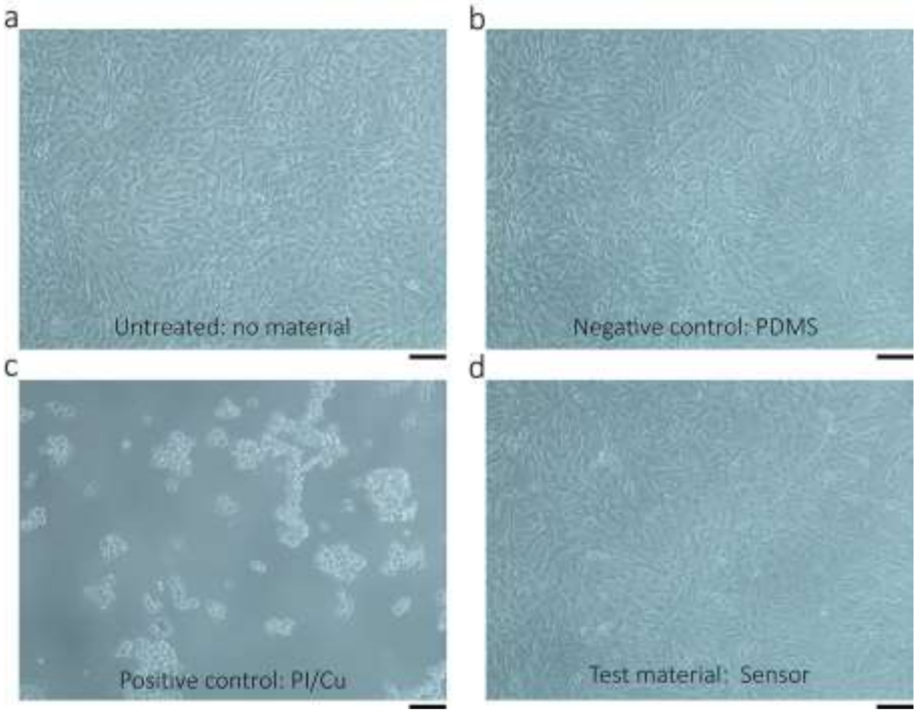

Figure S7 Direct contact cell cytotoxicity assay. Micrographs show representative cell (NIH/3T3 mouse embryonic fibroblast cells) in each culture flask after ~ 24 incubation with the indicated materials: **(a)** Untreated: no material; **(b)** Negative control: PDMS; **(c)** Positive control: PI/Cu; **(d)**Test material: Sensor. All images were taken at the same magnification (scale bars 100 µm)**.**

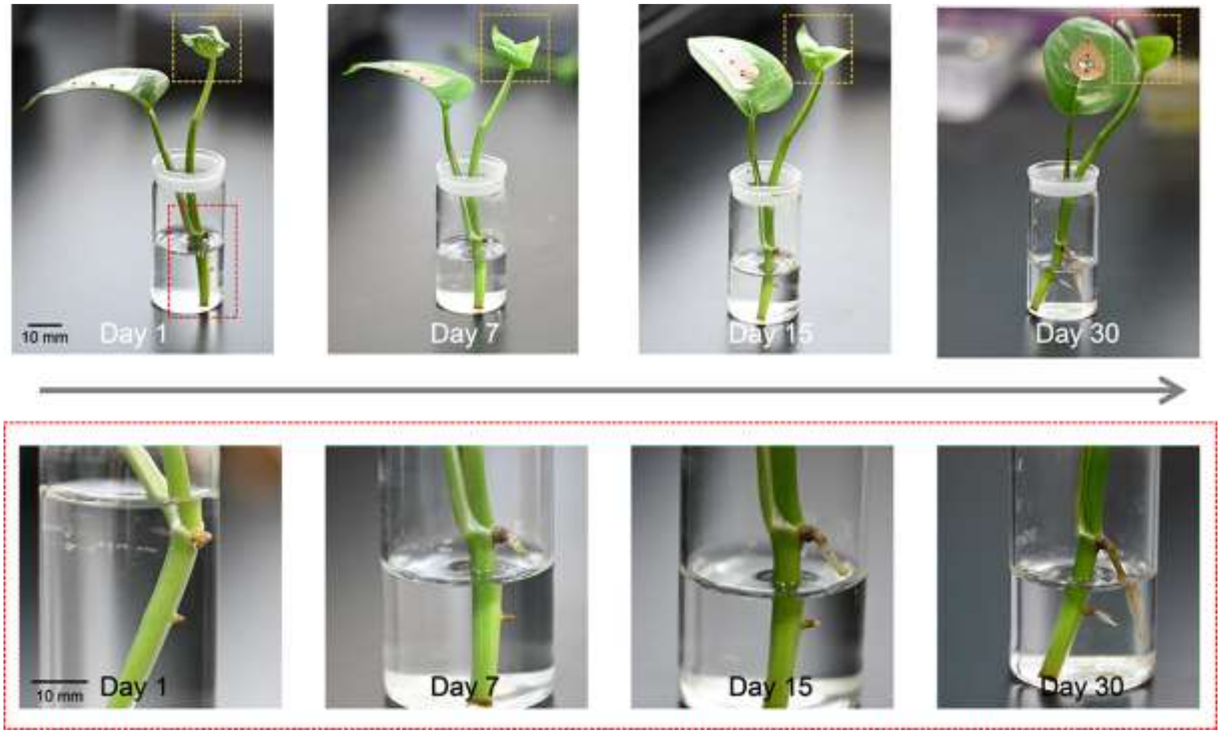

Figure S8 Optical images of a sensor-mounted pothos seedling after 30 days of indoor growth in culture solution (DEWODUO, China) under moderate sunlight. A small-sized seedling (height 15.5 cm) was selected and grown in our laboratory-scale reactor. After 30 days of growth, the seedling exhibited clear signs of health and vitality. The whole seedling remained green, and a new leaf was growing normally (yellow box). Besides, intensive root growth was observed (red box)

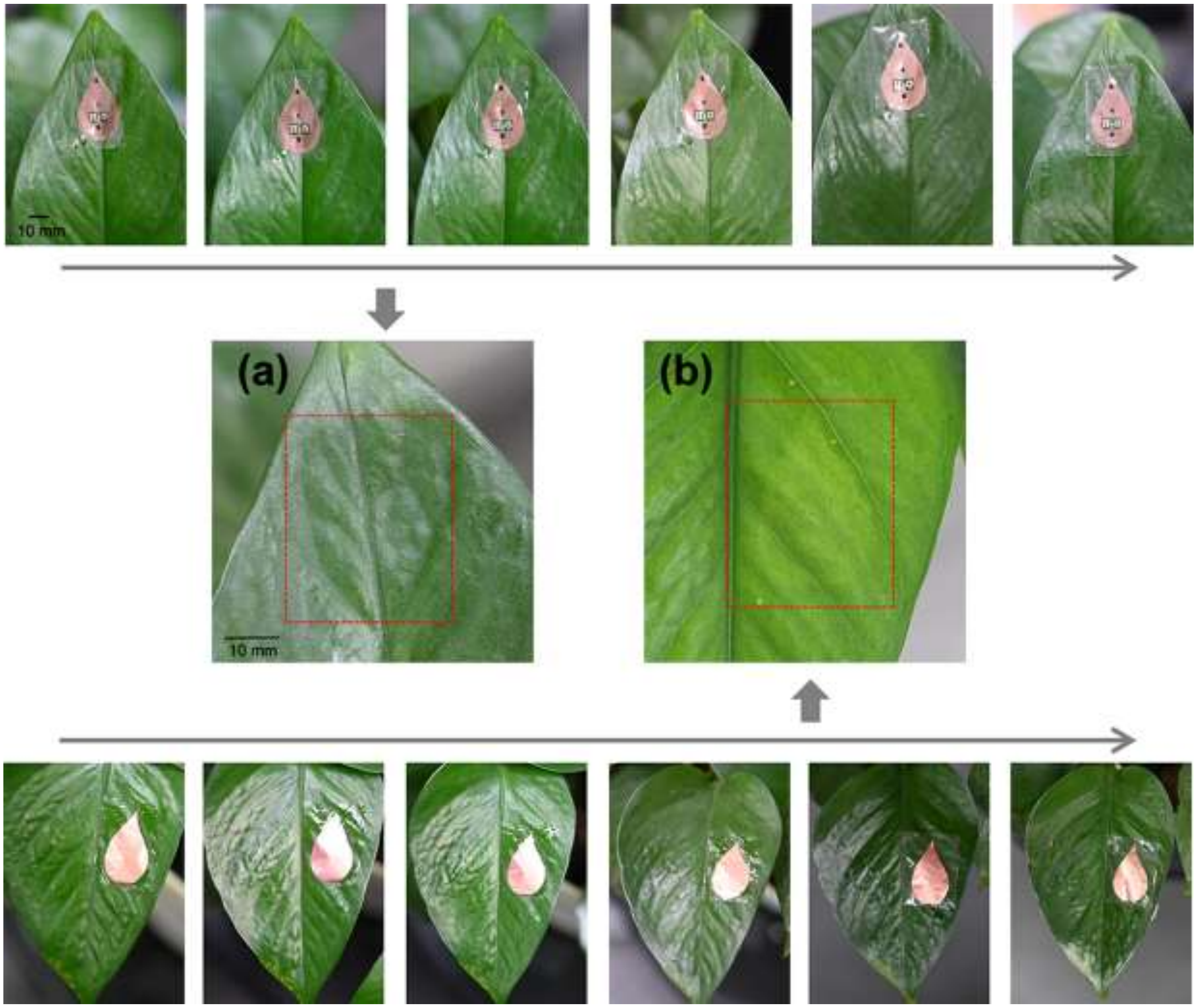

Figure S9 (a) Optical images of the sensor-mounted area on a pothos leaf after 100 days of in-door growth in soil under moderate sunlight. The area maintained healthy green in color, indicating that chlorophyll production was at a normal level. These observations suggested that the leaf's sensor-mounted area was able to uptake air, water, and nutrients and maintain vitality to the same degrees as the unmounted leaf. **(b)** On the contrary, when a Cu film (resembling the traditional type sensor) was also mounted on the leaf as a control group, the mounted area turned yellow, indicating a decrease in chlorophyll content, possibly due to lack of light or oxygen.

5. Calibration of the sensor.

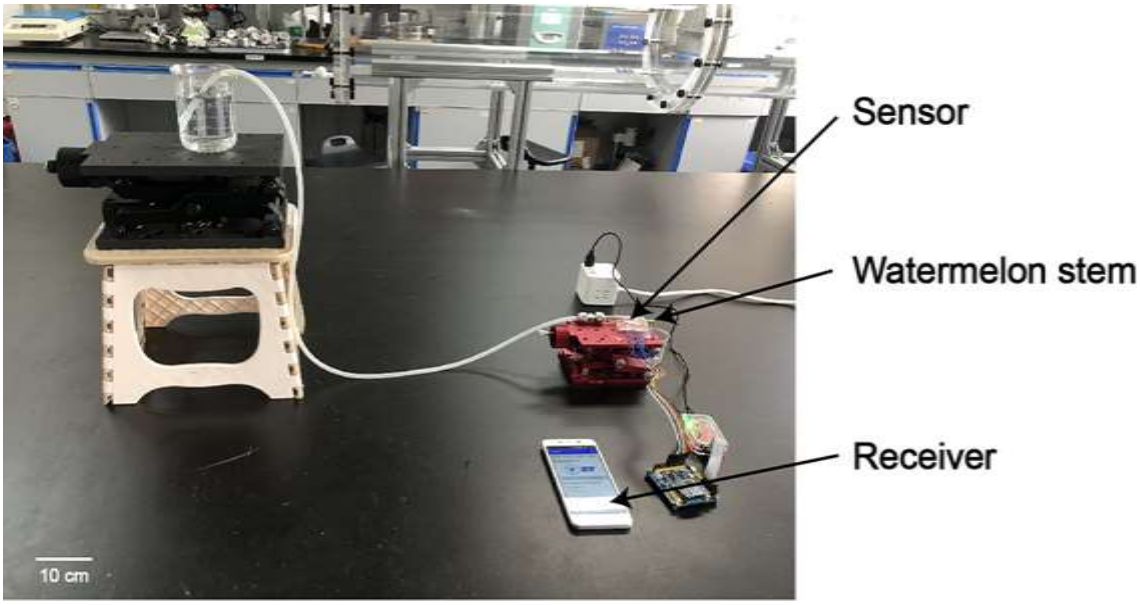

Figure S10 Experimental setup for calibration. A fresh-cut watermelon stem (length: 15mm； diameter：~ 4mm) was connected to a peristaltic pump with a stainless steel ferrule, and a rubber tube after a sap flow sensor was mounted in the middle of the stem. By adjusting the pump pressures, different sap flow rates could be obtained. The water flow through the stem was carefully collected and weighted by a balance to verify the exact flow rates.

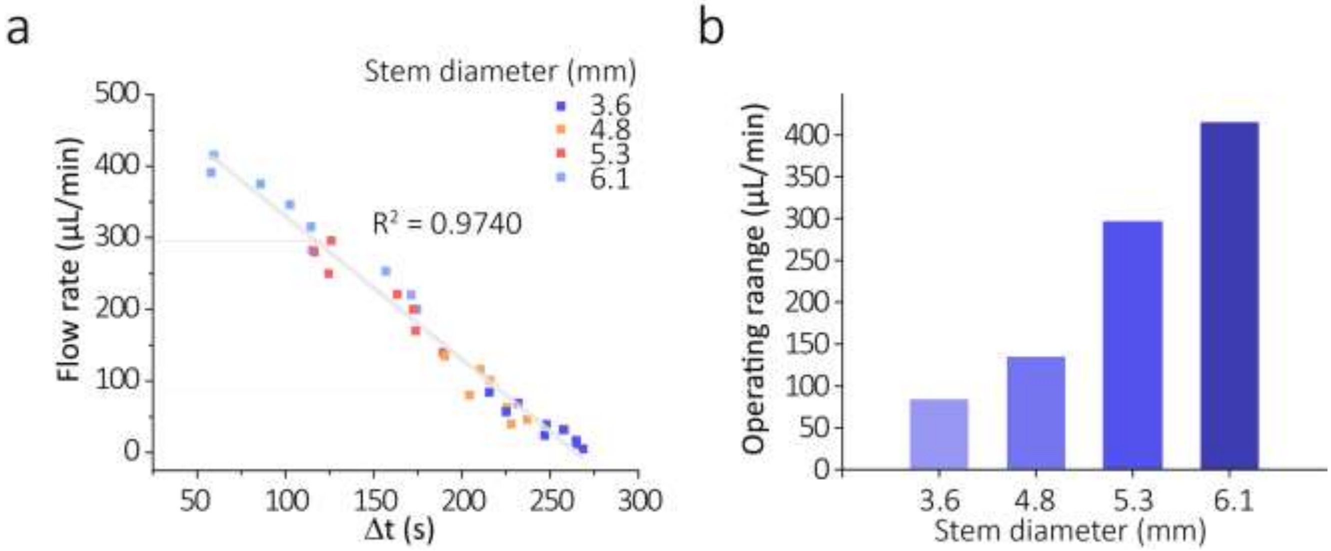

Figure S11 (a) Response of the sensor on the stems with different diameters. A single liner fit could be obtained for the flow rate (*R*) and time (Δt), indicating the sensor performance is not affected by the

stem's diameter. **(b)** However, our sensor's operating rages on various stems are different because the maximum water transport capacity for the different stem is different.

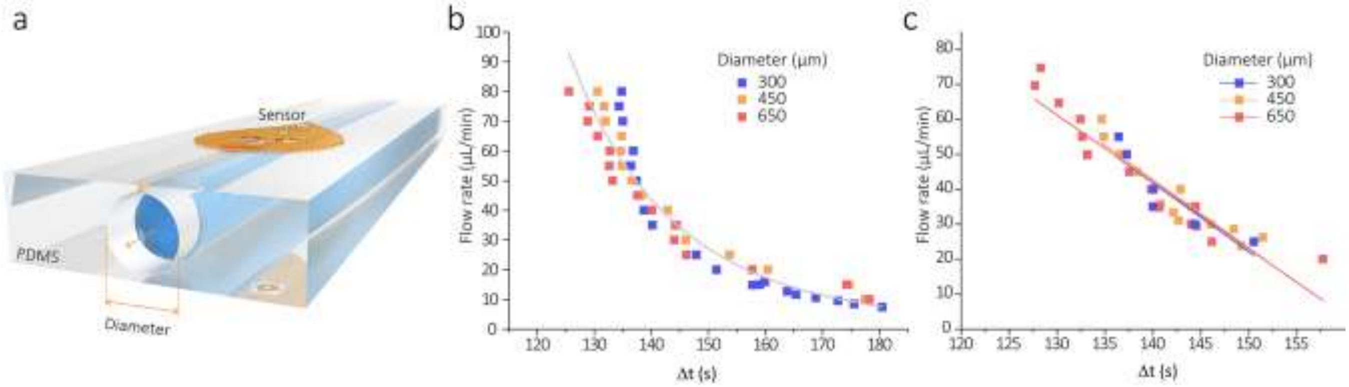

Figure S12 Effect of the vascular dimension on the measurement (a) Schematic illustration of the experiment setup. To understand the effect of vascular size, PDMS microchannels with defined dimensions (300–650 μm) were used to simulated the plant stem with different vascular tissue. One of our flow sensors was deployed on top of the PDMS microchannels. The channel wall thickness is about 1 mm. **(b)** The obtained Δt resulting from various flow rates (R). **(c)** Linear working curves obtained from corresponding data sets, which are almost identical, indicating the influence of vascular dimension on the measurement is not significant.

6. In-field Measurements.

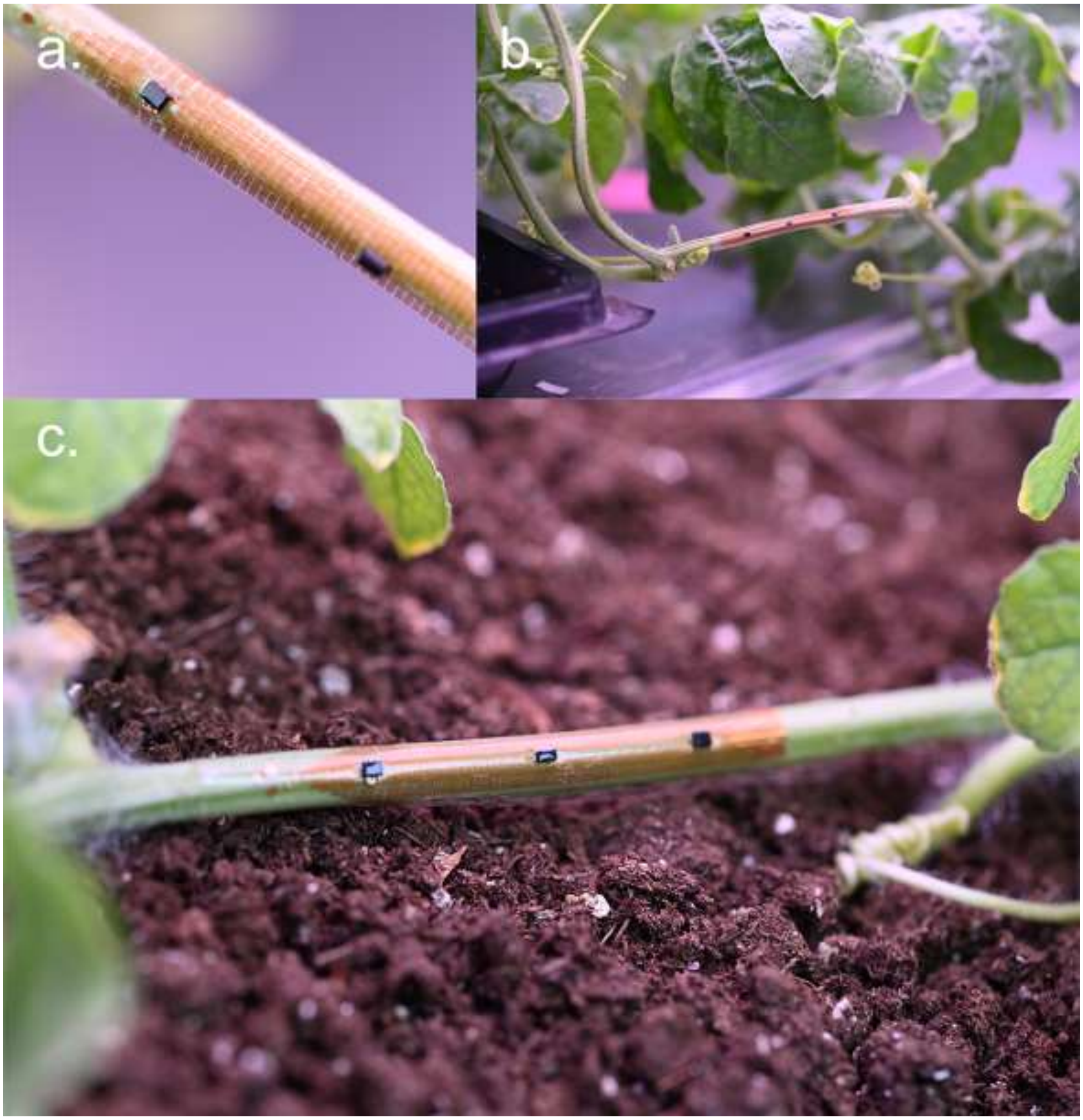

Figure S13 In-field monitor of sap flow rate. A healthy watermelon plant during maturation was selected for the study. The plant was grown in a farm field under ambient conditions. A sensor was mounted on a root stem with a diameter of ~ 4 mm. The experiment began shortly before sunrise. For each measurement, the thermistor heated for a short period (120 s), and the resulting temperature variations downstream and upstream were recorded during the followed 360 s. A total of ~ 5 min required for each measurement. To see the low-level sap fluctuation, the monitor was performed continuously for 18 h, between (05:00-23:00).

6.1 Study of the responses of watermelon to various environmental stresses.

(1) Temperature, humidity, illumination intensity

A healthy watermelon plant in soil was placed in an artificial climate chamber (temperature 25℃, relative humidity 80%, illumination intensity 24000 lux) after a sensor was mounted on the root stem about ~ 4 mm diameter. Then, the sap flow rate was measured under various (25℃, 30℃, 35℃), humidity level (40%, 90%), and illumination intensity (9600 – 28800 lux). The plant was kept undisturbed for at least 2 h before the measurement under each condition. Each measurement was repeated 3 times.

(2) Soil moisture level

A healthy watermelon plant in soil with a root stem of about ~ 4 mm was selected for this study. The plant was kept in-door for 2 days without watering. Then, the sap flow rate was determined before and after watering (15 min later). Each measurement was repeated 3 times.

7. Study of water distribution of watermelon

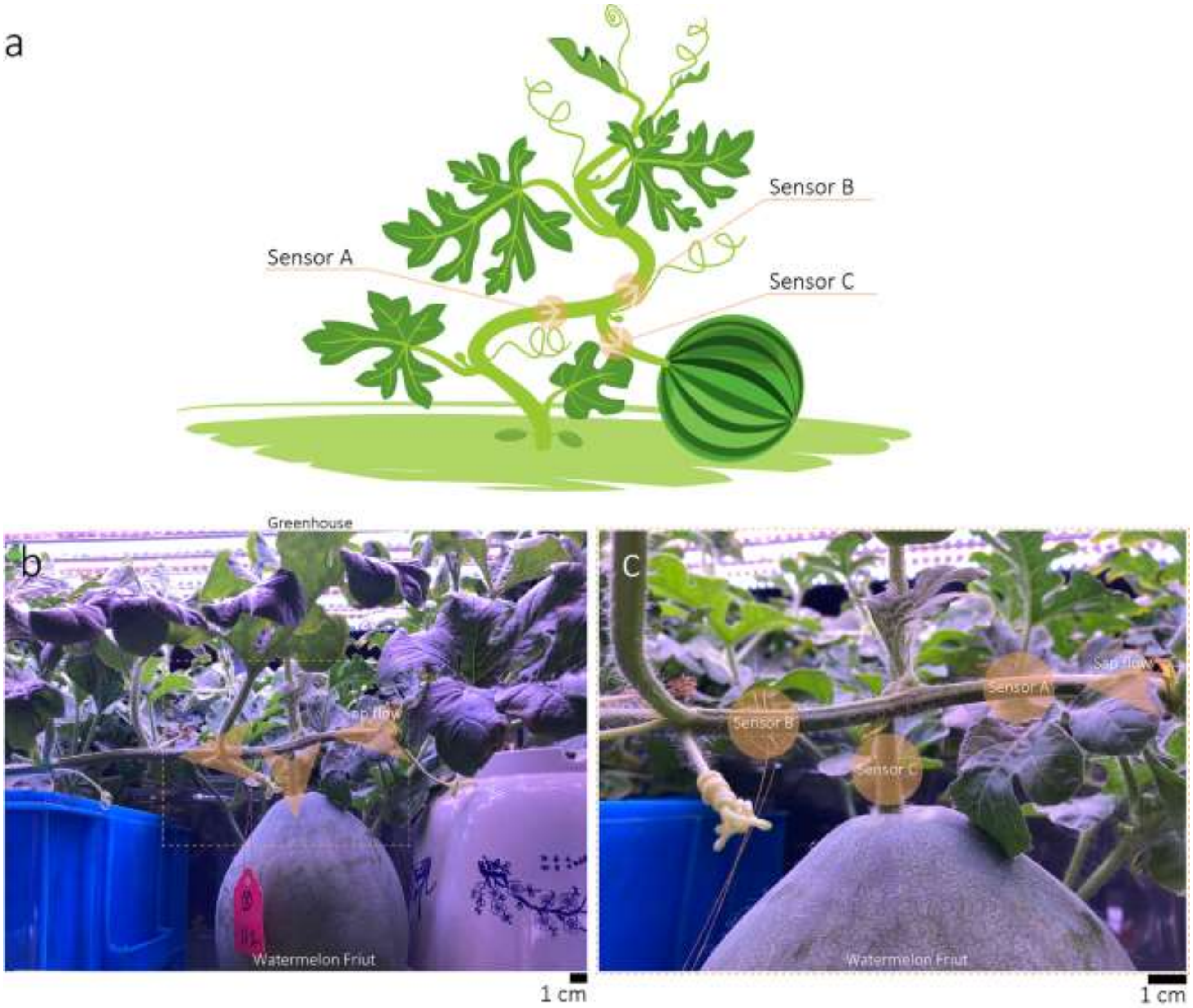

Figure S14 (a) Schematic illustration of the sensors' deployment (Sensor A: basal stem; Sensor B: apical branch; Sensor C: fruit branch). **(b-c)** Optical images of the watermelon plant used in this study. The diameter of the stem is about 5.8 mm.

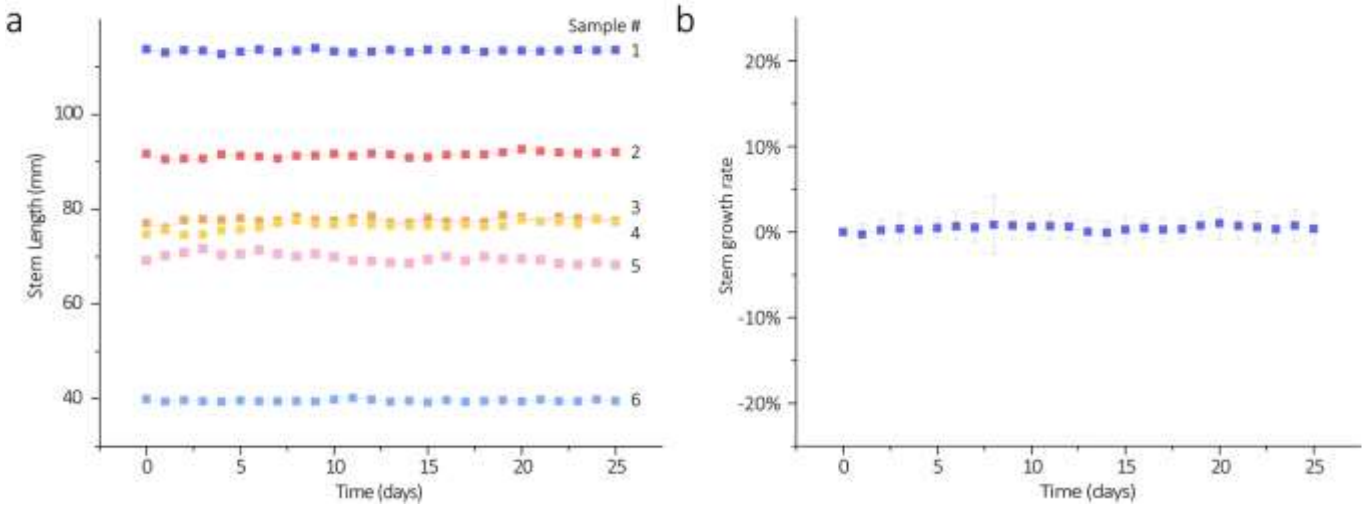

Figure S15 Watermelon stem growth rate. (a) Length of six watermelon stems in a month and **(b)** Averaged the watermelon plant stem's growth rate. Error bar: ±S.D.
